# Ultrasound Pattern of Indeterminate Thyroid Nodules with Prevalence of Oncocytes

**DOI:** 10.3390/jcm14155206

**Published:** 2025-07-23

**Authors:** Sium Wolde Sellasie, Stefano Amendola, Leo Guidobaldi, Francesco Pedicini, Isabella Nardone, Tommaso Piticchio, Simona Zaccaria, Luigi Uccioli, Pierpaolo Trimboli

**Affiliations:** 1 Division of Endocrinology and Diabetes, CTO Andrea Alesini Hospital, Department of Surgical Sciences, University of Rome Tor Vergata, 00153 Rome, Italy; 2 Ph.D. School of Applied Medical-Surgical Sciences, University of Rome Tor Vergata, 00133 Rome, Italy; 3 UOC of Pathologic Anatomy and Cytodiagnostic, Sandro Pertini Hospital, 00157 Rome, Italy; 4 Thyroid Endocrine Surgery, Sant’Eugenio Hospital, 00144 Rome, Italy; 5 Department of Medicine and Surgery, University Kore of Enna, 94100 Enna, Italy; 6 Faculty of Biomedical Sciences, Universitàdella Svizzera Italiana (USI), 6900 Lugano, Switzerland; 7 Clinic for Endocrinology and Diabetology, Ente Ospedaliero Cantonale, 6500 Bellinzona, Switzerland

**Keywords:** oncocytes, indeterminate thyroid nodules, ultrasound, ACR TI-RADS, cytology

## Abstract

**Objectives**: Oncocyte-rich indeterminate thyroid nodules (O-ITNs) present diagnostic and management challenges due to overlapping features between benign and malignant lesions and differing cytological classifications. This study aimed primarily to assess the ultrasound (US) characteristics and US-based risk of O-ITNs using the American College of Radiology Thyroid Imaging Reporting And Data Systems (ACR TI-RADS). A secondary objective was to compare the Bethesda System for Reporting Thyroid Cytopathology (BSRTC) and Italian Consensus for the Classification and Reporting of Thyroid Cytology (ICCRTC) cytological systems regarding classification and clinical management implications for O-ITNs. **Methods**: A retrospective study was conducted on 177 ITNs (TIR3A and TIR3B) evaluated between June 2023 and December 2024 at CTO-Alesini, Rome (Italy). Nodules were assessed with US, cytology, and histology. Oncocyte predominance was defined as >70% oncocytes on fine-needle aspiration (FNA). US features were analyzed according to ACR TI-RADS. Nodules were reclassified by BSRTC, and potential differences in clinical case management (CCM) were analyzed. **Results**: O-ITNs comprised 47.5% of the sample. Compared to non-O-ITNs, O-ITNs were larger and more frequently showed low-risk US features, including a higher prevalence of ACR TI-RADS 3 nodules. However, no progressive increase in the risk of malignancy (ROM) was observed across ACR TI-RADS classes within O-ITNs. Histological malignancy was identified in 47.1% of O-ITNs, a lower proportion compared to non-O-ITNs, though the difference was not statistically significant. Classification discordance with potential management impact was lower in O-ITNs (20.2%) than in non-O-ITNs (38.7%). **Conclusions**: O-ITNs typically exhibit benign-appearing US features and lower classification discordance between BSRTC and ICCRTC, yet US risk stratification fails to differentiate malignancy risk within O-ITNs. A tailored approach integrating cytology and cautious US interpretation is essential for optimal O-ITN management.

## 1. Introduction

Oncocytes, previously known as Hürthle cells, are large follicular cells with a granular eosinophilic cytoplasm. This characteristic granular appearance is due to the abundance of mitochondria. The accumulation of mitochondria in the cytoplasm is thought to result from changes in mitochondrial DNA (mtDNA), including point mutations and large deletions, which were initially detected in early molecular studies of oncocytic neoplasms [1].

These mitochondrial alterations may lead to impaired oxidative phosphorylation and ATP synthesis, which in turn could induce a compensatory proliferation of mitochondria [2]. Oncocytes are commonly found in both benign conditions, such as adenomas or thyroiditis, and malignant conditions, including thyroid carcinoma. Therefore, the presence of oncocytes presents a diagnostic challenge for thyroid specialists, as they are frequently observed in fine-needle-aspiration (FNA) specimens and are currently considered “atypical cells”.

This issue becomes even more concerning when oncocytes are predominant and present in the background of indeterminate cytological nodules (ITNs), which include both low-risk indeterminate (TIR3A) and high-risk indeterminate (TIR3B) categories in the Italian Consensus for the Classification and Reporting of Thyroid Cytology (ICCRTC) [3]. Specifically, “sparsely cellular samples containing predominantly microfollicular groups, also with oxyphilic features (‘Hürthle cells’), in a background of scant colloid, can fulfill the criteria for inclusion in the TIR3A category.” Conversely, samples composed exclusively or almost exclusively of oncocytes (“oncocytic neoplasm”) may be diagnosed as TIR3B. In the latest version of the Bethesda System for Reporting Thyroid Cytopathology (BSRTC) [4], oncocyte-rich ITNs can be categorized under the Atypia of Undetermined Significance (AUS) group, specifically as AUS-n when mild nuclear atypia is present or AUS-other (AUS-o) in its absence. Additionally, in the follicular neoplasm (FN) group, the specific designation FN-ONC is used when the sample is predominantly composed of oncocytes.

The differences between these two systems could prompt thyroidologists to treat ITNs rich in oncocytes differently, depending on the indications provided by the chosen reporting system. The management of ITNs rich in oncocytes is further complicated by the limitations of ancillary techniques, making ultrasound (US) evaluation a pivotal tool in these cases. However, few studies have focused on the US assessment of ITNs rich in oncocytes.

Given these diagnostic challenges, the primary objective of this study was to evaluate oncocyte-rich ITNs using US, specifically assessing their US-based risk. As a secondary objective, the study compared the two main cytological classification systems—namely, the BSRTC and the ICCRTC—in the evaluation of oncocyte-rich ITNs. To this end, a large cytological database of ITNs was retrospectively reviewed.

## 2. Materials and Methods

### 2.1. Institutional Setting and Management of ITNs

At the Endocrinology Unit of the CTO Hospital (Rome, Italy), patients with thyroid nodule(s) are evaluated by US during their clinical examination. After their initial assessment, patients undergo laboratory tests (mainly TSH-reflex and calcitonin). The indication for FNA is usually established according to American College of Radiology Thyroid Imaging Reporting And Data Systems [5] assessment and additional factors included in these guidelines (i.e., nodule size, operator experience, or patient anxiety), or in accordance with clinical indication posed by health care providers (i.e., endocrinologists, surgeons, otorhinolaryngologists, nuclear medicine specialists, or general practitioners). Once FNA is performed, the cytological smears are read and classified according to the ICCRTC [3]. The latter includes seven categories: inadequate (TIR1), cystic (TIR1C), not neoplastic (TIR2), low-risk (TIR3A) and high-risk (TIR3B) indeterminate lesions, suspicious for (TIR4) and consistent with (TIR5) malignancy. Apart from the US and FNA results, patients are generally managed according to individual clinical data, and the decision-making includes case discussion in the institutional multidisciplinary team when appropriate.

### 2.2. Case Selection

The study period was June 2023 to December 2024. From our outpatient database, patients who underwent diagnostic workup for thyroid nodules, with an indeterminate cytologic report according to ICCRTC (i.e., TIR3A or TIR3B), and further total or partial thyroidectomy were selected. Inclusion criteria were as follows: (I) age > 18 years; (II) detailed preoperative thyroid US report performed by skilled and experienced endocrinologist; (III) preoperative thyroid FNA; (IV) total or partial thyroidectomy performed in our Thyroid Surgery Unit; and (V) availability of a histological diagnosis in our pathology database. Patients undergoing diagnostic or therapeutic procedures at other institutions were excluded. All patients included in the present study gave informed consent and signed privacy forms.

### 2.3. Measures and Reference Standard

Oncocyte predominance was defined as when oncocytes represented more than 70% of cells in the FNA smear. Accordingly, the series was divided into two subgroups as oncocyte predominance-ITN (O-ITNs) or not (non-O-ITNs). In addition, other cytological features on FNA smears were evaluated and categorized as follows: (I) nuclear alterations: absent, minor atypia (including anisonucleosis), and major atypia; (II) scant/absent colloid (yes/no); (III) microfollicular arrangement (yes/no); and (IV) high cellularity (yes/no). To better explore the matter, the cases series was reclassified according to the BSRTC by an expert cytopathologist (L.G.). Then, considering the different structures and recommendations between ICCRTC and BSRTC, eventual changes in clinical case management (CCM) were analyzed.

Regarding US assessment, the ITNs were classified across the categories of ACR TI-RADS, as it is the most popular US-based risk-stratification system and because this is a point-based system [6] that is very suitable for studies. Accordingly, all ACR TI-RADS descriptors of its five issues were analyzed: (I) composition (cystic or almost completely cystic, spongiform, mixed cystic and solid, solid or almost completely solid), (II) echogenicity (anechoic, hyperechoic or isoechoic, hypoechoic, or very hypoechoic), (III) shape (wider than tall or taller than wide), (IV) margin (smooth, ill-defined, lobulated or irregular, or extra-thyroidal extension), and (V) echogenic foci (none or large comet-tail artifacts, macrocalcifications, peripheral rim calcifications, or punctate echogenic foci). Additionally, information on the nodule location within the thyroid was recorded: right or left lobe (upper, middle, or lower third) or isthmus.

Histological examination, if available, was the reference standard to calculate the risk of malignancy (ROM).

### 2.4. Statistical Analysis

Continuous variables were expressed as median and interquartile range (IQR) and compared with the Mann–Whitney U test. Other data were expressed as frequencies and compared using the chi-square test. A *p* value of <0.05 was considered to define statistical significance. All statistical analyses were performed with Jamovi software version 2.3 retrieved from https://www.jamovi.org.

## 3. Results

### 3.1. Case Study

The study series included a total of 177 ITNs from 176 patients (female 74.6%, median age 58 years, and IQR 49–65) (Table 1). The median maximum diameter of the ITNs was 15 mm (IQR 10–20 mm). Among them, 47 patients underwent thyroid surgery, and histological analysis revealed malignancy in 51.1%: twenty-two were papillary thyroid carcinomas (PTCs), one oncocytic carcinoma (OTC), and one medullary thyroid carcinoma (MTC). The remaining twenty-three patients with benign histology included eight oncocytic adenomas, five follicular adenomas, and ten follicular hyperplasias.

### 3.2. US Assessment

Regarding the US presentation, ITNs predominantly exhibited a solid or nearly solid composition (96%), hypoechoic echogenicity (58.8%), smooth margins (93.2%), a wider-than-tall shape (94.9%), and an absence of suspicious echogenic foci (83.1%). Based on these characteristics, most nodules were classified as TI-RADS 4 (61%) or TI-RADS 3 (26%) according to the ACR TI-RADS system. Additionally, the majority of nodules were located in the thyroid lobes (92.1%), with a predominant localization in the lower third (39.6%) (Table 1).

### 3.3. Cytological Assessment

According to the ICCRTC, ITNs were classified as TIR3A in 72.3% of cases and TIR3B in 27.7%. Conversely, based on the BSRTC, the distribution of cytological reports was as follows: AUS-n (42.4%), AUS-o (35.6%), FN (17.5%), and SFM (4.5%). In terms of pathological features, nuclear alterations such as minor atypia and major atypia were found in 68.9% and 20.9% of cases, respectively. Colloid was scant/absent in 16.9%, and microfollicular rearrangement was observed in 32.8%. Additionally, the smears showed high cellularity and were rich in oncocytes in 47.5% of cases (Table 1).

### 3.4. Differences Between O-ITN and Non-O-ITN

As summarized in Table 2, the ITN cohort was divided based on the presence of oncocyte predominance. No significant differences were found in epidemiological characteristics, specifically age and sex.

Regarding the US assessment, larger nodules, isoechoic or hyperechoic echogenicity, smooth margins, a wider-than-tall shape, and the absence of suspicious echogenic foci were more frequently observed in the O-ITN group compared to the non-O-ITN group. No significant differences were found in terms of composition or in thyroid location or position within the lobe. Consequently, the distribution of ACR TI-RADS categories differed significantly between the two subgroups, with a higher prevalence of ACR TI-RADS 3 and a lower prevalence of ACR TI-RADS 5 nodules observed in the O-ITN group compared to the non-O-ITN group (Table 2).

Among the cytologic reports (Table 2), a significantly different distribution of frequencies was observed between the ICCRTC classes; in fact, the O-ITN subgroup was predominantly composed of TIR3A cases, whereas the non-O-ITN subgroup showed a higher prevalence of TIR3B. This result was further supported by the single pathological characteristic analysis: in the O-ITN subgroup, nuclear alterations such as minor atypia and high cellularity were significantly more prevalent. In contrast, major atypia, absent or scant colloid, and a microfollicular arrangement were more commonly observed in the non-oncocytic ITNs.

Among the available histological data, although the non-O-ITN subgroup showed a higher malignancy rate, the difference in cancer rates between the O-ITN and non-O-ITN subgroups, as defined by the ROM, was not statistically significant (Table 2).

**Figure 1:**
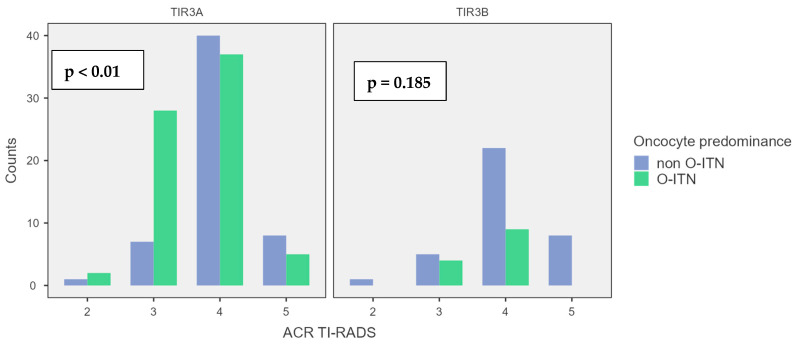
ACR TI-RADS distribution in ITNs according to ICCRTC.

Combining the US and cytological data, as detailed in Figure 1, in the US assessment using ACR-TIRADS, a statistically significant difference in ACR classification was observed for TIR3A nodules (*p* < 0.01) when oncocyte predominance, encountered in 56.3% of cases, was present. Specifically, this subgroup showed an increased number of nodules classified as having lower US risk (ACR TI-RADS 2–3). Conversely, for TIR3B nodules, oncocyte predominance, encountered in 26.5% of cases, did not significantly influence the US evaluation (*p* = 0.185). However, among the O-ITN, the US assessment comparing TIR3A and TIR3B did not show a statistically significant difference (*p* = 0.57).

To better explore these findings, the sensitivity, specificity, and accuracy of each US characteristic and ACR TI-RADS category in detecting O-ITNs were calculated, as detailed in Table 3. Once again, isoechogenicity—and consequently the ACR TI-RADS 3 category—demonstrated the highest accuracy, although it remained modest, reaching approximately 60%.

Combining the US and histological data, an increasing trend in the ROM was observed among non-O-ITNs, rising from 25% in ACR TI-RADS 3 to 60% in ACR TI-RADS 5. Conversely, all carcinomas among O-ITNs were classified as ACR TI-RADS 3 or 4, with ROMs of 40% and 55.6%, respectively.

### 3.5. Translation from ICCRTC to Other Cytological Classification Systems

Once ITNs were reclassified according to BSRTC, a significantly different distribution of frequencies was observed between the BSRTC classes (*p* < 0.01); in fact, the O-ITN subgroup was predominantly composed of AUS cases, whereas the non-O-ITN subgroup showed a higher prevalence of FN and SFM. Combining cytological and ACR TI-RADS data, we observed a consistently higher prevalence of lower ACR TI-RADS classes in the O-ITN subgroup compared to the non-O-ITN subgroup, reaching the statistical significance only in AUS nodules (*p* < 0.01). However, among the O-ITN, the US assessment comparing AUS, FN, and SFM did not show a statistically significant difference (*p* = 0.78).

The difference between the two cytological systems in classifying O-ITN and non-O-ITN was evaluated, using CCM as the outcome. For O-ITN cases, 20.2% showed a discordant diagnosis between ICCRTC and BSRTC that could lead to a change in management. Specifically, 69.2% of TIR3B O-ITN cases were classified as AUS, while 11.1% of TIR3A O-ITN cases were classified as FN.

In contrast, among non-O-ITN cases, 38.7% showed a discordant diagnosis with a potential impact on management: 58.3% of TIR3B non-O-ITN cases were classified as AUS, and 21.4% of TIR3A non-O-ITN cases were classified as FN/SFM. This difference in management impact between the two subgroups was statistically significant (*p* < 0.01).

## 4. Discussion

The identification of oncocytes in FNA often presents a diagnostic challenge for cytologists and clinical thyroidologists, particularly when encountered in the setting of an indeterminate cytological report. In such cases, US may play a crucial role in the management, but limited data are available in this specific setting. In addition, the presence of oncocytes can be variably classified both within a single cytological reporting system and across different systems, leading to divergent clinical management strategies depending on the classification system employed. This study aims to address this gap by firstly evaluating the US characteristics of O-ITNs using the ACR TI-RADS and secondly by comparing the classification outcomes of the two main cytological systems: the BSRTC and the ICCRTC.

The results of this study can be summarized as follows: First, among all the ITNs examined, O-ITNs—despite their larger size—were most frequently classified as being low risk based on the US assessment (ACR TI-RADS 3). However, when stratified by cytological class, the ACR TI-RADS evaluation did not effectively distinguish between the different cytological categories of O-ITNs, and ROM did not show a progressive increase across ACR TI-RADS classes. Second, when comparing the two cytological systems using CCM as the outcome, the classification of O-ITNs appeared to be more consistent between the two systems than for non-O-ITNs. These findings warrant a clinically oriented and thorough discussion.

**Figure 2:**
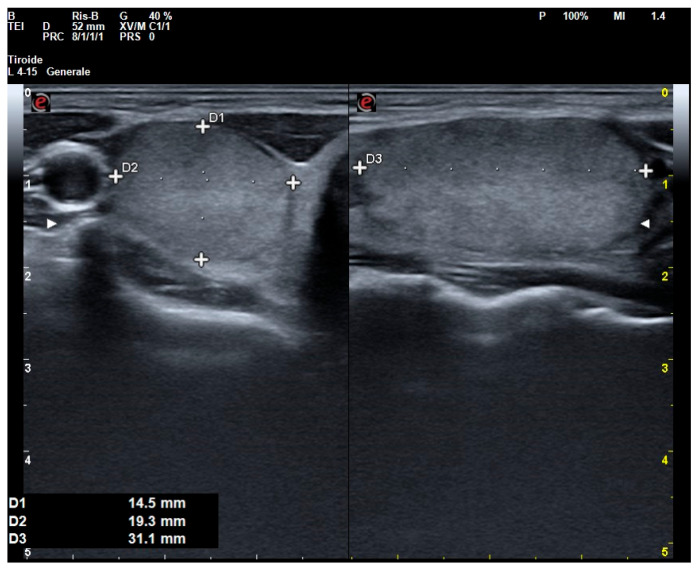
US presentation of O-ITN, with anteroposterior (D1), lateral (D2), and longitudinal (D3)diameters, classified as ACR TI-RADS 3, with a TIR3A cytologic report and a proven histology of oncocytic adenoma.

Regarding US assessment, O-ITN appears to carry a lower US-adjusted risk, but only when it is classified within the lowest risk category of cytological systems among ITNs (TIR3A and AUS). In fact, O-ITNs were more frequently solid, isoechoic rather than hypoechoic, with a wider-than-tall shape and a smooth margin, and typically lacked high-risk echogenic foci (Figure 2). As a result, these nodules were most often classified as ACR TI-RADS 3. This category, in fact, seems to have greater but limited accuracy in identifying O-ITNs. The findings of this study emphasize that our current understanding of US patterns associated with O-ITNs remains incomplete. Nonetheless, the results indicate that when a nodule is solid and isoechoic, exhibits smooth margins, and lacks high-risk US features, the probability of it being an O-ITN is not negligible. Should these lower-risk US characteristics be validated and expanded upon by larger, prospective studies, they may serve as the foundation for the development of a dedicated US-based risk-stratification system, thereby preserving the central role of US in the clinical management of thyroid nodules.

According to the ACR TI-RADS white paper [5], FNA is recommended for ACR TI-RADS 3 nodules only if the maximum diameter exceeds 25 mm. Although the median diameter of the ITNs included in this study was below that threshold, O-ITNs were found to be significantly larger than other ITNs. Therefore, it is reasonable to assume that O-ITNs may more frequently meet the size criterion for FNA, in line with ACR TI-RADS and also the other US risk-stratification systems. To further complicate this issue, it is important to highlight the increasing incidence of thyroid incidentalomas, which is partly attributable to the widespread use of PET-FDG. In many cases, the observed focal and increased uptake is associated with thyroid nodules rich in oncocytes; these nodules are often further evaluated by FNA, frequently leading to a diagnosis of O-ITN [7].

The progressive growth pattern potentially linked to mitochondrial impairment observed in O-ITNs [8] may prompt clinicians to repeat FNA, often leading to confirmed indeterminate cytological results without a clear management pathway [9]. For example, according to current European guidelines [10], the diagnostic workup of an O-ITN appearing as a solid isoechoic nodule without high-risk ultrasound features—classified as EU-TIRADS 3 and accompanied by a previous AUS cytology report—may range from ultrasound follow-up to surgery. This diagnostic uncertainty is further compounded by the high prevalence of O-ITNs among ITNs in daily clinical practice—approximately 50% in our study—which contrasts sharply with the lower prevalence rates reported in current guidelines [3,4]. Therefore, since O-ITNs are mostly assessed as lower-risk on US, it is reasonable to avoid repeating FNA in cases of a TIR3A cytological diagnosis, provided that no US changes occur during follow-up, as already recommended in the current guidelines [9,10].

Given these considerations, optimizing the diagnostic workup of O-ITNs is crucial to reduce both healthcare costs and patient anxiety. While ancillary techniques currently have limitations, US continues to play a central and effective role in the assessment and management of these lesions. However, the inability of US to distinguish risk-stratification differences among O-ITNs classified within the various categories of the indeterminate cytological system represents a significant diagnostic limitation, reflecting the point of weakness of the US risk-stratification systems, tailored mainly to the PTC [11]; in fact, the majority of O-ITN lacks high-risk and papillary-like US features such as marked hypoechogenicity, microcalcifications, irregular shape, or margins. Similar results, with a different study design, were observed by another previous study [12], in which another risk-stratification system, such as EU TI-RADS [13], was inefficient in distinguishing between benign and malignant nodules. In addition, within the O-ITN group, there were no cases of malignancy classified as ACR TI-RADS 5. Most carcinomas were assessed as ACR TI-RADS 4, and to a lesser extent as ACR TI-RADS 3. This result could be partly attributed to the presence of non-papillary malignancies—such as MTC and OTC—which are not accurately evaluated by US [11,14]. Furthermore, there was a high prevalence of the follicular subtype of PTC, which is recognized as having a lower US risk profile compared to the classic subtype [15,16].

It should be noted that, among the ITNs that underwent thyroid surgery, the ROM of O-ITNs was lower than that of non-O-ITNs (47.1% vs. 53.3%, respectively), although this difference did not reach statistical significance. Therefore, the presence of oncocytes in the FNA smear does not appear to increase the risk of malignancy in ITNs. This finding is consistent with previous studies that included all BSRTC categories and assessed the extent of oncocyte presence in relation to histologic outcomes [12,17,18].

A secondary aim of this study was to examin the differing interpretations of ITN smears according to the ICCRTC and BSRTC systems. Notably, in both subcategories of the two systems (TIR3A/AUS and TIR3B/FN), the management recommendations for ITNs were similar. For the lower-risk categories, a conservative approach is generally preferred, involving repeat FNA or US follow-up. In contrast, for TIR3B/FN cases, a surgical approach may be considered.

Surprisingly, although the cytological features suggestive of PTC should have consistently influenced both systems, there was greater concordance between the two systems among the O-ITNs. This resulted in a statistically significant lower CCM, accounting for 20% of cases—approximately half the rate observed in non-O-ITNs. The high number of TIR3B/AUS cases in both subgroups (i.e., O-ITN and non-O-ITN) may reflect the central point of divergence between the BSRTC and ICCRTC: the classification of nuclear atypia [19]. Although this was revised in the latest version of the BSRTC [4], its management remained unchanged. Particularly, in our cohort, minor atypia was notably more prevalent among O-ITNs, whereas major atypia was more commonly observed in non-O-ITNs. These two subgroups exhibited distinct patterns of cytomorphologic alterations: O-ITNs were characterized by minor nuclear atypia and high cellularity, while non-O-ITNs more frequently displayed a microfollicular arrangement, scant colloid, and major atypia. This divergence in cytological features may reflect underlying differences in the molecular and genomic profiles of O-ITNs compared to non-O-ITNs [14,20]. In particular, the molecular basis of oncocytic change involves alterations in mitochondrial DNA, including copy number variations. Only in malignant cases do additional molecular alterations—such as mutations in genes like RAS or TERT, which also act as drivers in non-oncocytic ITN neoplasms—tend to occur [20,21].

This study has several limitations that should be acknowledged. First, its retrospective, single-center design and small sample size—particularly concerning the histologic data—limit the generalizability of the findings. Therefore, these results should be validated by larger, prospective, multicenter studies. Second, given the complexity and variability in the management of ITNs, the results regarding malignancy risk and histological outcomes may be affected by selection bias. This is particularly relevant for ITNs with the lowest cytological risk in which surgery is typically reserved for selected cases and is often not primarily driven by a suspicion of malignancy. The study cohort included only nodules with TIR3A/TIR3B cytology during the clinical practice, which were subsequently reclassified according to the BSRTC. While this may represent a potential selection bias, the rationale lies in the fact that this system (ICCRTC) is used in our routine clinical practice. Moreover, the two ICCRTC subcategories (TIR3A and TIR3B) have demonstrated superior diagnostic accuracy, with statistically significant differences in cancer rates, as reported in the literature [19]. Despite these limitations, the exploratory nature of this study should be emphasized. Its primary contribution lies in the US assessment of O-ITNs using the ACR TI-RADS, with both cytology and histology as reference standards. Additionally, the study provides a comparative analysis of the two major cytological classification systems for O-ITNs, which may aid in improving the clinical management of these lesions.

## 5. Conclusions

O-ITNs represent a significant subset of ITNs and warrant a standardized diagnostic and therapeutic approach. US evaluation using ACR TI-RADS identified O-ITNs as lower risk compared to non-O-ITNs. However, it was not effective in stratifying risk within the O-ITN group based on cytological classification or histological outcomes, highlighting a key diagnostic limitation of US. To date, no US-related characteristics can independently or significantly impact the management of O-ITNs, which always requires the addition of clinical and cytological data. In this context, the two main cytological systems—BSRTC and ICCRTC—despite their acknowledged differences, appeared more concordant in classifying O-ITNs than non-O-ITNs. As a result, the management of O-ITNs seems to be more similar across the two cytological systems investigated.

## Figures and Tables

**Table 1:** Descriptive analysis of the sample included in the study.

Demographic Features	
Cases, n	177
Age, years (IQR)	58.0 (49.0–65.0)
Gender (male; female), %	25.4; 74.6
Nodule’s US data	
Maximum ITN diameter, mm (IQR)	15.0 (10.0–20.0)
ACR TI-RADS (1; 2; 3; 4; 5), %	0.0; 1.1; 26.0; 61.0; 11.9
Composition (cystic or almost completely cystic, spongiform, mixed cystic and solid, or solid or almost completely solid), %	0.0; 0.0; 4.0; 96.0
Echogenicity (anechoic, hyperechoic or isoechoic, hypoechoic, or very hypoechoic), %	0.0; 35.0; 58.8; 6.2
Shape (wider than tall or taller than wide), %	94.9; 5.1
Margin (smooth, ill-defined, lobulated or irregular, or extra-thyroidal extension), %	93.2; 0.0; 6.8; 0.0
Echogenic foci (none or large comet-tail artifacts, macrocalcifications, peripheral rim calcifications, or punctate echogenic foci), %	83.1; 11.3; 4.0; 1.6
Lobe (right; left; isthmus), %	44.9; 47.2; 8.0
Position in the lobe (upper; middle; lower), %	30.7; 29.7; 39.6
Nodule’s cytological characterization	
Cytological classification-ICCRTC (TIR3A; TIR3B), %	72.3; 27.7
Cytological classification-BSRTC (AUS-n; AUS-o; FN; SFM), %	42.4; 35.6; 17.5; 4.5
Nuclear alterations (absent; minor atypia; major atypia), %	10.2; 68.9; 20.9
Scant/absent colloid (yes; no), %	16.9; 83.1
Oncocyte predominance (yes; no), %	47.5; 52.5
Microfollicular arrangement (yes; no), %	32.8; 67.2
High cellularity (yes; no), %	48.0; 52.0

Data expressed as medians and IQR or proportions (percentage) as appropriate. US: ultrasound; ITN: indeterminate thyroid nodule; ACR TI-RADS: American College of Radiology Thyroid Imaging Reporting And Data Systems; ICCRTC: Italian Consensus for the Classification and Reporting of Thyroid Cytology; TIR3A: low-risk indeterminate lesion; TIR3B: high-risk indeterminate lesion; BSRTC: Bethesda System for Reporting Thyroid Cytopathology; AUS-n: nuclear Atypia of Undetermined Significance; AUS-o: other Atypia of Undetermined Significance; FN: follicular neoplasm; SFM: Suspicious for Malignancy.

**Table 2:** Descriptive and comparative analysis (non-O-ITN vs. O-ITN) of ITNs enrolled.

	Non-O-ITN (n = 92)	O-ITN (n = 85)	*p*-Value
Age, years (IQR)	58.0 (48–64)	59.0 (50–67)	0.53
Gender (male; female), %	22.8; 77.2	28.2; 71.8	0.41
Maximum ITN diameter, mm (IQR)	14.0 (9.7–19.3)	16.0 (11–23)	0.04
ACR TI-RADS classes (1; 2; 3; 4; 5), %	0.0; 2.2; 13; 67.4; 17.4	0.0; 2.4; 37.6; 54.1; 5.9	<0.01
Composition (cystic or almost completely cystic, spongiform, mixed cystic and solid, or solid or almost completely solid), %	0.0; 0.0; 3.3; 96.7	0.0; 0.0; 4.7; 95.3	0.62
Echogenicity (anechoic, hyperechoic or isoechoic, hypoechoic, or very hypoechoic), %	0.0; 26.1; 66.3; 7.6	0.0; 44.7; 50.6; 4.7	0.03
Shape (wider than tall or taller than wide), %	91.3; 8.7	98.8; 1.2	0.02
Margin (smooth, ill-defined, lobulated or irregular, or extra-thyroidal extension), %	89.1; 0.0; 10.9; 0.0	97.6; 0.0; 2.4; 0.0	0.02
Echogenic foci (none or large comet-tail artifacts, macrocalcifications, peripheral (rim) calcifications, or punctate echogenic foci), %	75.0; 17.4; 5.4; 2.2	91.8; 4.7; 2.4; 1.2	0.03
Lobe (right; left; isthmus), %	45.1; 49.5; 5.5	44.7; 44.7; 10.6	0.44
Position in the lobe (upper; middle; lower), %	38.0; 26.0; 36.0	41.2; 33.3; 25.5	0.49
Cytological classification-ICCRTC (TIR3A; TIR3B), %	60.9; 39.1	84.7; 15.3	<0.01
Cytological classification-BSRTC (AUS-n; AUS-o; FN; SFM), %	15.2; 55.4; 23.9; 5.4	71.8; 14.1; 10.6; 3.5	<0.01
Nuclear Alterations (absent; minor atypia; major atypia), %	8.7; 59.8; 31.5	11.8; 78.8; 9.4	<0.01
Scant/absent colloid (yes; no), %	27.2; 78.8	5.9; 94.1	<0.01
Microfollicular arrangement (yes; no), %	57.6; 42.4	5.9; 94.1	<0.01
High cellularity (yes; no), %	25.0; 75.0	72.9; 27.1	<0.01
ROM, %	53.3	47.1	0.67

Data expressed as medians (i.q. range) or proportions (percentage) as appropriate. Mann–Whitney U test for continuous variables or chi-square test for discrete variables, as appropriate. O-ITN: indeterminate thyroid nodule with oncocyte predominance; ACR TI-RADS: American College of Radiology Thyroid Imaging Reporting And Data Systems; ICCRTC: Italian Consensus for the Classification and Reporting of Thyroid Cytology; TIR3A: low-risk indeterminate lesion; TIR3B: high-risk indeterminate lesion; BSRTC: Bethesda System for Reporting Thyroid Cytopathology; AUS-n: nuclear Atypia of Undetermined Significance; AUS-o: other Atypia of Undetermined Significance; FN: follicular neoplasm; SFM: Suspicious for Malignancy; ROM: risk of malignancy.

**Table 3:** Sensitivity, specificity, and accuracy in detecting O-ITN of US characteristics and ACR TI-RADS.

US	Sensitivity, %	Specificity, %	Accuracy, %
Composition			
Mixed cystic and solid	4.8	96.4	53.1
Solid or almost completely solid	95.2	3.2	46.9
Echogenicity			
Hyperechoic or isoechoic	45.2	74.2	60.5
Hypoechoic	50	33.3	41.2
Markedly hypoechoic	4.8	92.5	50.8
Shape			
Wider than tall	98.8	8.6	51.6
Taller than wide	1.2	91.4	48.6
Margin			
Smooth	98.8	10.8	52.5
Lobulated or irregular	1.2	89.2	47.5
Echogenic foci			
None or large comet-tail artifacts	91.7	24.7	56.5
Macrocalcifications	4.8	82.8	45.8
Peripheral rim calcifications	2.4	94.6	50.8
Punctate echogenic foci	1.2	97.8	52
ACR TI-RADS			
2	2.4	97.8	52.5
3	38.1	87.1	63.8
4	53.6	32.3	42.4
5	6.0	82.8	46.3

Data expressed as percentage. US: ultrasound; O-ITN: indeterminate thyroid nodule with oncocyte predominance; ACR TI-RADS: American College of Radiology Thyroid Imaging Reporting And Data Systems.

## Data Availability

The datasets generated during and/or analyzed during the current study are available from the corresponding author upon reasonable request.

## Abbreviations

The following abbreviations are used in this manuscript:

ITN	Indeterminate thyroid nodule
O-ITN	Oncocyte-rich indeterminate thyroid nodule
US	Ultrasound
ICCRTC	Italian Consensus for the Classification and Reporting of Thyroid Cytology
BSRTC	Bethesda System for Reporting Thyroid Cytopathology
PTC	Papillary thyroid carcinoma
MTC	Medullary thyroid carcinoma
OTC	Oncocytic thyroid carcinoma

## References

[1] Wong K.S., Angell T.E., Barletta J.A., Krane J.F. (2021). Hürthle cell lesions of the thyroid: Progress made and challenges remaining. Cancer Cytopathol..

[2] Máximo V., Soares P., Lima J., Cameselle-Teijeiro J., Sobrinho-Simões M. (2002). Mitochondrial DNA Somatic Mutations (Point Mutations and Large Deletions) and Mitochondrial DNA Variants in Human Thyroid Pathology. Am. J. Pathol..

[3] Nardi F., Basolo F., Crescenzi A., Fadda G., Frasoldati A., Orlandi F., Palombini L., Papini E., Zini M., Pontecorvi A. (2014). Italian consensus for the classification and reporting of thyroid cytology. J. Endocrinol. Investig..

[4] Ali S.Z., Baloch Z.W., Cochand-Priollet B., Schmitt F.C., Vielh P., VanderLaan P.A. (2023). The 2023 Bethesda System for Reporting Thyroid Cytopathology. Thyroid.

[5] Tessler F.N., Middleton W.D., Grant E.G., Hoang J.K., Berland L.L., Teefey S.A., Cronan J.J., Beland M.D., Desser T.S., Frates M.C. (2017). ACR Thyroid Imaging, Reporting and Data System (TI-RADS): White Paper of the ACR TI-RADS Committee. J. Am. Coll. Radiol..

[6] Tappouni R.R., Itri J.N., McQueen T.S., Lalwani N., Ou J.J. (2019). ACR TI-RADS: Pitfalls, Solutions, and Future Directions. RadioGraphics.

[7] Poller D.N., Megadmi H., Ward M.J.A., Trimboli P. (2020). Hürthle Cells on Fine-Needle Aspiration Cytology Are Important for Risk Assessment of Focally PET/CT FDG Avid Thyroid Nodules. Cancers.

[8] Schatz-Siemers N., Brandler T.C., Oweity T., Sun W., Hernandez A., Levine P. (2019). Hürthle cell lesions on thyroid fine needle aspiration cytology: Molecular and histologic correlation. Diagn. Cytopathol..

[9] Piticchio T., Sellasie S.W., D’aRrigo F., Galeano F., Barca I., Prinzi A., Le Moli R., Scappaticcio L., Amendola S., Guidobaldi L. (2024). Clinical management of indeterminate thyroid nodules needs to be revisited. New evidence for a personalized approach to the problem. J. Endocrinol. Investig..

[10] Durante C., Hegedüs L., Czarniecka A., Paschke R., Russ G., Schmitt F., Soares P., Solymosi T., Papini E. (2023). 2023 European Thyroid Association Clinical Practice Guidelines for thyroid nodule management. Eur. Thyroid J..

[11] Matrone A., Gambale C., Biagini M., Prete A., Vitti P., Elisei R. (2021). Ultrasound features and risk stratification systems to identify medullary thyroid carcinoma. Eur. J. Endocrinol..

[12] Słowińska-Klencka D., Wysocka-Konieczna K., Klencki M., Popowicz B. (2020). Usability of EU-TIRADS in the Diagnostics of Hürthle Cell Thyroid Nodules with Equivocal Cytology. J. Clin. Med..

[13] Russ G., Bonnema S.J., Erdogan M.F., Durante C., Ngu R., Leenhardt L. (2017). European Thyroid Association Guidelines for Ultrasound Malignancy Risk Stratification of Thyroid Nodules in Adults: The EU-TIRADS. Eur. Thyroid J..

[14] Kure S., Ohashi R. (2020). Thyroid Hürthle Cell Carcinoma: Clinical, Pathological, and Molecular Features. Cancers.

[15] Sparano C., Rotondi M., Verdiani V., Brunori P., Castiglione F., Bartoli C., Perigli G., Badii B., Vezzosi V., Simontacchi G. (2022). Classic and Follicular Variant of Papillary Thyroid Microcarcinoma: 2 Different Phenotypes Beyond Tumor Size. J. Endocr. Soc..

[16] Tallini G., Tuttle R.M., Ghossein R.A. (2017). The History of the Follicular Variant of Papillary Thyroid Carcinoma. J. Clin. Endocrinol. Metab..

[17] Ren Y., Kyriazidis N., Faquin W.C., Soylu S., Kamani D., Saade R., Torchia N., Lubitz C.C., Davies L., Stathatos N. (2020). The Presence of Hürthle Cells Does Not Increase the Risk of Malignancy in Most Bethesda Categories in Thyroid Fine-Needle Aspirates. Thyroid.

[18] Wong K.S., Jo V.Y., Lowe A.C., Faquin W.C., Renshaw A.A., Shah A.A., Roh M.H., Stelow E.B., Krane J.F. (2020). Malignancy risk for solitary and multiple nodules in Hürthle cell–predominant thyroid fine-needle aspirations: A multi-institutional study. Cancer Cytopathol..

[19] Trimboli P., Ferrarazzo G., Cappelli C., Piccardo A., Castellana M., Barizzi J. (2022). Thyroid Nodules with Indeterminate FNAC According to the Italian Classification System: Prevalence, Rate of Operation, and Impact on Risk of Malignancy. An Updated Systematic Review and Meta-analysis. Endocr. Pathol..

[20] Bischoff L.A., Ganly I., Fugazzola L., Buczek E., Faquin W.C., Haugen B.R., McIver B., McMullen C.P., Newbold K., Rocke D.J. (2024). Molecular Alterations and Comprehensive Clinical Management of Oncocytic Thyroid Carcinoma. JAMA Otolaryngol. –Head Neck Surg..

[21] Abi-Raad R., Prasad M.L., Adeniran A.J., Cai G. (2022). Copy number variations identified in thyroid FNA specimens are associated with Hürthle cell cytomorphology. Cancer Cytopathol..

